# Supplementary material for: Turbulent mass transfer caused by vortex induced reconnection in collisionless magnetospheric plasmas
Source: Nat Commun. 2017 Nov 17;8:1582. doi: 10.1038/s41467-017-01579-0 (PMC5693928; doi:10.1038/s41467-017-01579-0)
Supplement: Supplementary file 1 — Supplementary Information [file 41467_2017_1579_MOESM1_ESM.pdf]

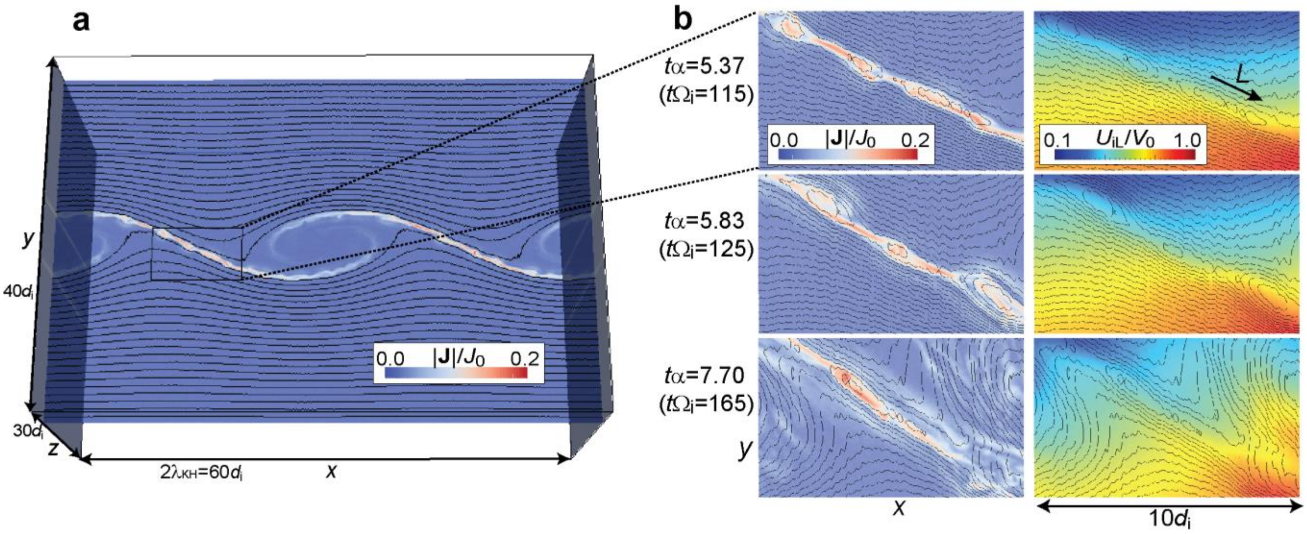

Supplementary Figure 1| Evolution of the vortex-induced reconnection within a symmetric small-scale system. **a**, 3D view of the amplitude of the current density |**J**| in an early non-linear growth phase of the KHI ($t\alpha$=5.37) in the smaller vortex (λ_{KH}=30d_i) with symmetric initial conditions (no plasma and field asymmetry across the boundary) described in Ref. [1]. **b**, Zoom-in views of the 2D contours in the x-y plane at z=23.4d_i of |**J**| and the ion bulk flow component U_{iL} along with the compressed current layer at $t\alpha$=5.37 and 5.83 (early non-linear phase), and $t\alpha$=7.70 (late non-linear phase). Black curves in **a**,**b** denote the in-plane magnetic field lines. The L-direction is parallel to the compressed current layer as indicated in the top panel in **b**. The plots in **b** demonstrate (i) that the system is too small to permit the development of ion-scale reconnection jets (i.e., no clear peak of U_{iL} component), (ii) that the size of the primary flux ropes in the L-direction is ~2-3d_i (~0.1λ_{KH}) and (iii) that in the early non-linear phase ($t\alpha$<6), these flux ropes preserve their structures without being disturbed by the secondary 3D tearing mode, while the structures become more turbulent in the later non-linear phase.

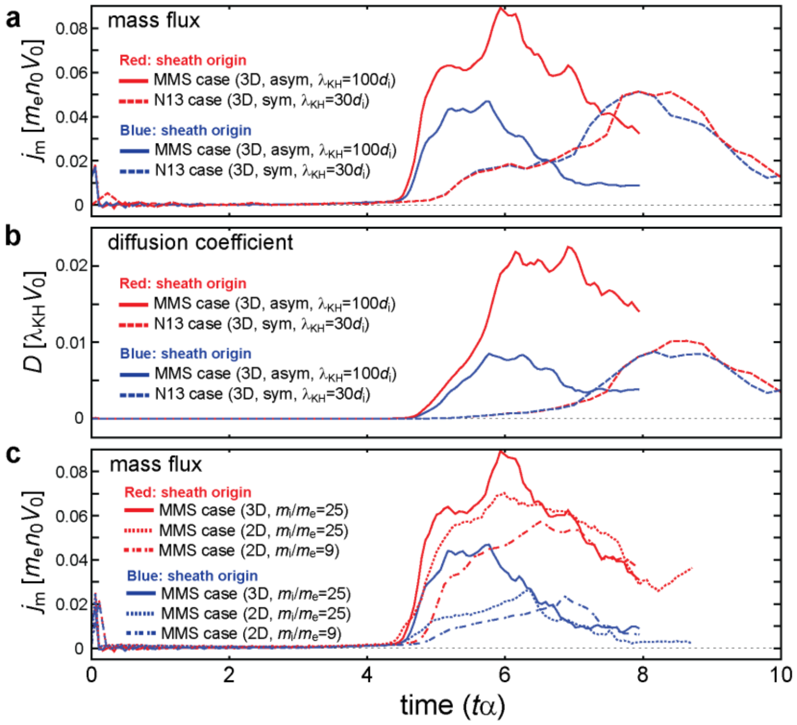

Supplementary Figure 2| Influence of ion to electron mass ratio (m_i/m_e), system size and dimensionality on the evolution of the plasma transport. a,b, Time evolution of (**a**) the mass flux j_m across the mixing surfaces defined by $|F_e|$=0.99 and (**b**) the corresponding diffusion coefficient D estimated from j_m/D~$m_e n_0/L_{mix}$ where L_{mix} is the averaged thickness of the mixing region in the y-direction. These panels contrast the large 3D simulation shown in the present letter (hereafter referred to as the large 3D simulation) with the smaller 3D simulation from Ref. [1]. **c**, Time evolution of the mass flux j_m, comparing the large 3D simulation with corresponding 2D simulations using the same initial basic conditions (but with m_i/m_e=25 and m_i/m_e=9). Although oblique tearing modes cannot occur in these 2D simulations, the primary ion-scale reconnection jets are reproduced within the restricted simulation plane. The plots **a** and **b** demonstrate that in the early non-linear phase for the large 3D simulation the mass flux and the diffusion coefficient are more than three to ten times larger than the smaller 3D simulation. Similarly, the mass transfer values from Ref. [2] in which λ_{KH}=15d_i are also much smaller than the large 3D simulation (not shown). Physically, this difference is due to the ion-scale jet formation and the secondary 3D turbulence that occurs only in the larger simulation. These figures also demonstrate that the transport quantities in the smaller-scale vortex is largely enhanced in the late non-linear phase ($t\alpha$>~7). This corresponds to the enhanced turbulence seen in the bottom plots of Supplementary Fig. 1b. As shown in panel **c,** the mass flux in the corresponding 2D cases, in which only the ion-scale jet formation occurs, is 30-50% smaller than the large 3D case, and does not depend strongly on m_i/m_e. Considering the difference (~0.03$m_e n_0 V_0$) of the mass fluxes between the early and late non-linear phases in the 3D smaller vortex case in **b** as well as the difference (~0.02$m_e n_0 V_0$) between the 2D and 3D cases in **c**, the turbulent development of the secondary 3D tearing mode contributes about 30-50% of the total flux.

SUPPLEMENTARY REFERENCES

[1] Nakamura, T. K. M., Daughton, W., Karimabadi, H., & Eriksson, S., Three-dimensional dynamics of vortex-induced reconnection and comparison with THEMIS observations, *Journal of Geophysical Research*, **118**, 5742-5757 (2013).

[2] Nakamura, T. K. M., & Daughton, W., Turbulent plasma transport across the Earth's low-latitude boundary layer, *Geophysical Research Letters*, **41**, 8704-8712 (2014).
